# CSF Inflammation Markers Associated with Asymptomatic Viral Escape in Cerebrospinal Fluid of HIV-Positive Individuals on Antiretroviral Therapy

**DOI:** 10.3390/v15091829

**Published:** 2023-08-29

**Authors:** Debjani Guha, Vikas Misra, Jun Yin, Dana Gabuzda

**Affiliations:** 1 Department of Cancer Immunology and Virology, Dana-Farber Cancer Institute, Boston, MA 02215, USA; 2 Department of Neurology, Harvard Medical School, Boston, MA 02115, USA

**Keywords:** HIV, central nervous system, cerebrospinal fluid, inflammation, neuroinflammation, IP-10, YKL-40

## Abstract

HIV establishes a viral reservoir in the CNS despite viral suppression in the blood on antiretroviral therapy (ART). In a minority of people with HIV (PWH), HIV RNA is detectable in CSF when HIV RNA in plasma is undetectable or HIV RNA levels are higher in CSF compared with plasma, an event termed CSF viral escape that can occur with or without neurological symptoms. Asymptomatic CSF viral escape occurs in 3–20% of PWH on ART, yet associated biomarkers are unclear. To identify biomarkers associated with asymptomatic CSF viral escape, we performed a matched group study of PWH on ART with vs. without CSF viral escape (n = 10 and n = 60, respectively, matched for age, duration of HIV infection, nadir CD4 count, and ART regimen) and 50 HIV-negative controls. PWH were on 3 or more ART drugs for >1 year, and the group with no CSF viral escape was suppressed below 50 copies/mL in plasma and CSF. Biomarkers of inflammation (IFN-γ, IL-1β, IL-6, IL-8, IL-15, IP-10, MCP-1, VEGF), cell adhesion (ICAM-1, VCAM-1), CNS injury (NFL), and glial activation (GFAP, YKL-40) were measured in paired plasma and CSF using the Meso Scale Discovery platform. PWH with vs. without CSF viral escape had more individuals (40%) with a plasma viral load (VL) > 50 copies/mL, higher CSF VL (median 156 vs. 40 copies/mL; *p* < 0.0001), lower CD4 count (318 vs. 512; *p* = 0.045), and higher CSF WBC (median [IQR] 4 [0–22] vs. 2 [0–4] cells/µL; *p* = 0.15) but similar proportions with HIV-associated neurocognitive disorders (HAND) (50% vs. 47%). CSF viral escape was associated with increased IL-1β, IFN-γ, IP-10, ICAM-1, and VCAM-1 in CSF but not plasma; IP-10 had the strongest association (*p* = 0.0008). CSF VL and WBC correlated with IFN-γ, IP-10, ICAM-1, and VCAM-1 (*p* < 0.05). Although markers of CNS injury showed no significant association with asymptomatic CSF viral escape, CSF YKL-40 correlated positively with CSF IL-1β (*p* = 0.003), IFN-γ (*p* = 0.0008), IP-10 (*p* < 0.0001), and NFL (*p* = 0.06) and negatively with neurocognitive T scores (*p* = 0.02). These findings identify CSF inflammation and glial activation markers that may serve as surrogate measures of HIV persistence in the CNS for future studies on therapeutics targeting the CNS reservoir.

## 1. Introduction

HIV infects the CNS and establishes a persistent viral reservoir in the brain despite viral suppression in the blood to below detectable levels on antiretroviral therapy (ART). HIV can be detected in cerebrospinal fluid (CSF) soon after acute infection, while early ART initiation has been shown to decrease the size of the brain viral reservoir and associated CNS injury [1,2]. Most HIV-infected cells in the brain are macrophages and microglia, but HIV+ CD4+ T cells also contribute to the CNS viral reservoir [1,3,4,5,6]. Successful HIV eradication and cure will require approaches that effectively target CNS viral reservoirs.

HIV-associated neurocognitive disorders (HAND), consisting of asymptomatic neurocognitive impairment (ANI), mild neurocognitive disorder (MND), and HIV-associated dementia (HAD), remain prevalent in people with HIV (PWH) despite viral suppression on ART [7,8]. Although the prevalence of HAD has declined due to the improved efficacy of ART, the prevalence of ANI and MND has increased [8]. The mechanisms underlying HAND are multifactorial, including effects of persistent CNS viral reservoirs, immune activation, neuroinflammation, ART drug neurotoxicity, vascular disease, and substance use disorder [3,7,8,9,10]. Biomarkers of monocyte activation and neuroinflammation, including elevated CSF neopterin, sCD14, sCD163, MCP-1, and IP-10, are frequently elevated in PWH on ART [11,12,13,14,15,16,17] and have been associated with a risk of neurocognitive impairment [3,8,18]. Biomarkers of CNS injury and glial activation, including neurofilament light chain (NFL), glial fibrillary acidic protein (GFAP), and YKL-40 (also known as Chitinase 3-like 1, CHI3L1), are also detected in a subset of PWH on ART, particularly in those with viremia [15,17,18,19,20,21,22,23]. The identification of biomarkers that can distinguish between biologically defined subtypes (biotypes) of HAND with different etiologies is important for evaluating new therapies and developing tailored interventions [3,17,24,25].

In most PWH on ART, HIV RNA is suppressed to below detectable levels in both plasma and CSF; when plasma HIV RNA is detectable, HIV RNA levels are typically more than one log10 copies/mL lower in CSF than in plasma [3,7,26,27,28]. However, in a minority of PWH, HIV RNA is detectable in CSF when HIV RNA in plasma is undetectable or HIV RNA levels are higher in CSF compared with plasma, an event termed CSF viral escape that can occur with or without neurological symptoms [14,29,30,31,32,33,34,35,36,37,38]. The prevalence of CSF viral escape regardless of neurological symptoms is estimated at 3–20% among PWH on ART [30,33,37,38,39,40,41]. Risk factors include low-level viremia, increased CSF white blood cell (WBC) count, low CD4 nadir, years of HIV infection and exposure to ART, low CNS penetration by some ART drugs, particularly atazanavir and other protease inhibitors, and drug-resistance mutations [14,34,35,36,37,38,39,42]. Asymptomatic CSF viral escape occurs in the absence of new or progressive neurological impairment and is typically detected when lumbar punctures are performed for research purposes [14,29,30,33,39]. In contrast, neurosymptomatic CSF viral escape, particularly in cases with high CSF HIV RNA levels > 1000 copies/mL, is often manifested by neurocognitive decline and white matter abnormalities on MRI imaging [31,33,34,38,40,41,43].

The identification of biomarkers associated with CSF viral escape is relevant for clinical management and developing strategies to achieve HIV cure. However, few reports have been published on biomarkers in CSF viral escape; limited data in these studies detected biomarkers of CNS immune activation and neuroinflammation, particularly neopterin, IP-10, and VCAM-1 [12,14,29,37,38,39]. Here, we performed a matched group study of PWH on ART with asymptomatic CSF viral escape and CSF HIV RNA > 50 copies/mL vs. viral suppression in plasma and CSF HIV RNA < 50 copies/mL to evaluate plasma and CSF biomarkers of neuroinflammation, glial activation, and CNS injury associated with asymptomatic CSF viral escape independent of HAND. We then evaluated the relationship between biomarker levels and HAND status.

## 2. Materials and Methods

### 2.1. Study Participants

Plasma and CSF samples from 120 individuals (n = 70 HIV+ individuals on ART and n = 50 HIV-controls) were collected between 2006 and 2016. HIV+ individuals were enrolled at four sites in the National NeuroAIDS Tissue Consortium (NNTC) (Manhattan HIV Brain Bank, National Neurological AIDS Bank, California NeuroAIDS Tissue Network, and Texas NeuroAIDS Research Center) and six sites in the CNS HIV Anti-Retroviral Therapy Effects Research (CHARTER) study (University of California San Diego, Johns Hopkins University, Baltimore, MD, Icahn School of Medicine at Mount Sinai, New York City, University of Texas Medical Branch, Galveston, University of Washington, Seattle, and Washington University, St. Louis, MO, USA). Recruitment procedures and eligibility criteria for NNTC and CHARTER have been described [44,45]. All individuals were enrolled with written informed consent and Institutional Review Board (IRB) approval at each study site. Samples and clinical data were collected and coded to protect participants’ confidentiality in accordance with IRB-approved protocols at the University of Texas Medical Branch Galveston, University of California Los Angeles, Icahn School of Medicine at Mount Sinai, University of California San Diego, Johns Hopkins University, University of Washington, Washington University, and Dana Farber Cancer Institute (DFCI protocol 16-273).

Eligible HIV+ participants were adults on a stable combination ART regimen including three or more ART drugs for ≥1 year with one or more paired plasma and CSF samples with viral load (VL) measurements between 2006–2016 and sufficient sample volumes available for the proposed studies. Lumbar punctures were performed for research purposes. Plasma and CSF VL were measured using assays considered to have a lower limit of detection of 50 HIV RNA copies/mL (Roche Amplicor Monitor v1.0/v1.5 or Abbott m2000 Real-Time assay); three CSF samples had additional available measurements to detect HIV RNA below 50 copies/mL using a single copy assay [16,26]. We excluded individuals with neuropsychological impairment due to other non-HIV-related causes, recent CNS infections, and CNS neoplasms. Asymptomatic CSF viral escape was defined as CSF greater than plasma HIV RNA or CSF HIV RNA detectable when plasma HIV RNA was undetectable in the absence of new neurological symptoms or discernable neurological decline. We included two cases that do not meet all eligibility criteria because a review of 135 plasma-CSF sample pairs identified only 8 asymptomatic CSF viral escape cases (5.9%): these two cases had plasma VL > 1000 and <10,000 copies/mL and CSF VL > 50 and <1000 copies/mL. HIV+ groups with CSF VL > 50 copies/mL vs. <50 copies/mL were matched for age, duration of HIV infection, nadir CD4 count, and type of ART regimen. We employed this matched-group study design to evaluate differences in biomarker levels while avoiding potentially confounding effects of variables that are known risk factors for CSF viral escape [14,34,35,36,38,39,42]. Plasma and CSF samples from HIV-negative control individuals without a diagnosed neurological disease (from Bioreclamation LLC, Westbury, New York, NY, USA) were group-matched for age, gender, and race.

### 2.2. Neurocognitive Testing and HAND Diagnoses

HIV+ individuals were administered a comprehensive neuropsychological test battery designed to assess seven neurocognitive domains (abstraction/executive function, speed of information processing, attention/working memory, learning, memory, verbal fluency, and motor function). Demographically corrected global neurocognitive T scores were generated from individual test T scores, as described [45,46]. T scores correlate negatively with the severity of neurocognitive impairment, with values below 40 (corresponding to 1 standard deviation of 10 below a normalized mean of 50) signifying impairment. HAND diagnoses were determined using established criteria [47] based on neurocognitive testing and neurological evaluation. PWH were classified as cognitively impaired if they had a HAND diagnosis of ANI or MND, corresponding to mild cognitive impairment (at least 2 neurocognitive domains with T score values below 40) without or with functional impairment in activities of daily living, respectively.

### 2.3. Meso Scale Discovery Assays

Plasma and CSF samples were centrifuged at 1200 rpm for 5 min at 4 °C, and the supernatants were aliquoted and stored at −80 °C. Plasma and CSF concentrations of inflammation markers (IFN-γ, IL-1β, IL-6, IL-8, IL-15, IP-10, MCP-1, VEGF), cell adhesion molecules (ICAM-1, VCAM-1), CNS injury marker (NFL), and glial activation markers (GFAP and YKL-40) were measured using the Meso Scale Discovery (MSD) platform (Rockville, MD) according to the manufacturer’s protocols and data analyzed using MSD discovery workbench 4.0 software, as described [48]. Detection limits for these biomarkers are listed at https://www.mesoscale.com/~/media/files/handout/assaylist.pdf, accessed on 26 August 2023.

### 2.4. Measurement of CSF/Plasma Albumin Ratio

CSF and plasma albumin levels were measured using the BCG albumin assay kit (Thermo Fisher Scientific, Waltham, MA, USA), as described [48]. The CSF-plasma albumin ratio was calculated as an indirect indicator of blood-brain barrier integrity.

### 2.5. Statistical Analysis

Demographics and clinical characteristics were compared between groups of interest by the nonparametric Mann–Whitney U test for continuous variables and the chi-square test or Fisher’s exact test for categorical variables. Differences in log10 transformed biomarker levels between paired plasma and CSF samples were analyzed by the paired Wilcoxon signed-rank test and between groups of interest by the Mann–Whitney U test. For these statistical analyses, a two-sided *p*-value < 0.05 was considered statistically significant (no Bonferroni correction was applied). Relationships between continuous variables were analyzed by Spearman’s rank correlation. Statistical analyses were performed using GraphPad Prism, version 9.0.

## 3. Results

### 3.1. Characteristics of the Study Cohort

The demographic and clinical characteristics of the study cohort are summarized in Table 1. The cohort included 70 HIV+ individuals on ART and 50 HIV-controls matched for demographics. Overall, HIV+ individuals were predominantly male (86%) and white (64%), with a median age 50 years (IQR, 46–55), a duration of HIV infection of 15 years, nadir CD4 count 65 cells/µL, and CD4 count 495 cells/µL. Compared with HIV+ individuals, HIV- individuals were of similar age, gender, and race. HIV+ individuals were grouped according to CSF VL < 50 HIV RNA copies/mL (n = 60) and >50 HIV RNA copies/mL (n = 10), matched for age, duration of HIV infection, nadir CD4 count, and type of ART regimen. HIV+ individuals with CSF VL > 50 copies/mL vs. <50 copies/mL had similar demographics, ART regimens (50% vs. 57% on protease inhibitors, 10% and 18% on integrase inhibitors, 30% and 42% on NNRTI, respectively), duration of ART treatment (median 8 vs. 10 years; *p* = 0.27), and CSF/plasma albumin ratio (6 vs. 4; *p* = 0.91), but they have a higher proportion of detectable plasma VL > 50 copies/mL (40% vs. 0%; *p* < 0.0001), a higher CSF VL (median 156 [IQR 65–1364] vs. 40 [40–40] copies/mL; *p* < 0.0001), a lower CD4 count (318 [200–532] vs. 512 [354–756]; *p* = 0.045), and an increasing trend for CSF white blood cell counts (median 4 [0–22] vs. 2 [0–4] cells/µL; *p* = 0.15). The most commonly used ART drugs were emtricitabine/tenofovir (53%), efavirenz (29%), 3TC (27%), ritonavir-boosted atazanavir (22%), abacavir (20%), and darunavir (19%); the most commonly used integrase inhibitor was raltegravir (14%). There were no significant differences between groups by CSF VL > 50 copies/mL vs. <50 copies/mL in percentage with a HAND diagnosis (ANI or MND) (50% vs. 47%, respectively), global neurocognitive T score (median 47 vs. 47), and BDI score (median 6 vs. 7).

### 3.2. Increased Inflammation Biomarkers and Cell Adhesion Molecules in CSF But Not Plasma of HIV+ Individuals on ART with CSF VL > 50 Copies/mL vs. <50 Copies/mL

**Figure 1:**
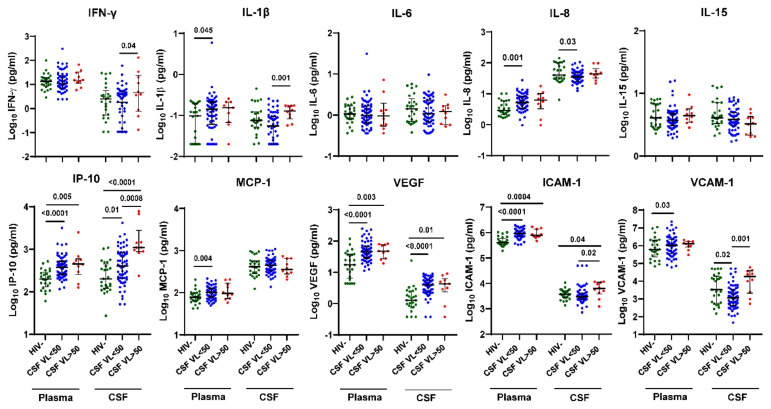
IFN-γ, IL-1β, IP-10, ICAM-1, and VCAM-1 levels are increased in CSF but not plasma of HIV+ individuals on ART with CSF VL > 50 copies/mL versus <50 copies/mL. Ten inflammation markers were measured in plasma and CSF from HIV+ individuals on ART (n = 60 with CSF VL < 50 copies/mL and n = 10 with CSF VL > 50 copies/mL) and HIV- controls (n = 25 plasma and n = 25 CSF) using the Meso Scale Discovery platform. Horizontal bars represent medians, vertical lines the interquartile range (IQR). *p*-values calculated using Mann-Whitney U test; significant differences (*p* < 0.05) are indicated.

Next, we compared plasma and CSF inflammation biomarker levels by HIV status and CSF VL > 50 copies/mL vs. <50 copies/mL (Figure 1). Compared with HIV- controls, plasma and CSF IP-10 and VEGF and plasma ICAM-1 levels were higher in both HIV+ individuals with CSF VL > 50 copies/mL (*p* = 0.005, <0.0001, 0.003, 0.01, 0.0004, respectively) and CSF VL < 50 copies/mL (*p* < 0.001, =0.01, <0.001, <0.001, <0.001), while plasma IL-1β, IL-8, MCP-1, and VCAM-1 were higher only in HIV+ individuals with CSF VL < 50 copies/mL (*p* = 0.045, 0.001, 0.004, 0.03, respectively). In contrast, plasma IFN-γ, IL-6, and IL-15 and CSF IL-6, IL-15, and MCP-1 levels were not significantly different between groups by HIV status. In HIV+ individuals with CSF VL > 50 copies/mL vs. <50 copies/mL, IFN-γ, IL-1β, IP-10, ICAM-1, and VCAM-1 levels were increased in CSF (*p* = 0.04, 0.001, 0.0008, 0.02, 0.001, respectively) but not plasma. Among these biomarkers, CSF IP-10 had the strongest association with CSF viral escape (*p* = 0.0008).

### 3.3. Inflammation Biomarkers in CSF of HIV+ Individuals on ART with Detectable CSF HIV RNA Correlate with CSF HIV RNA Levels and CSF WBC

**Figure 2:**
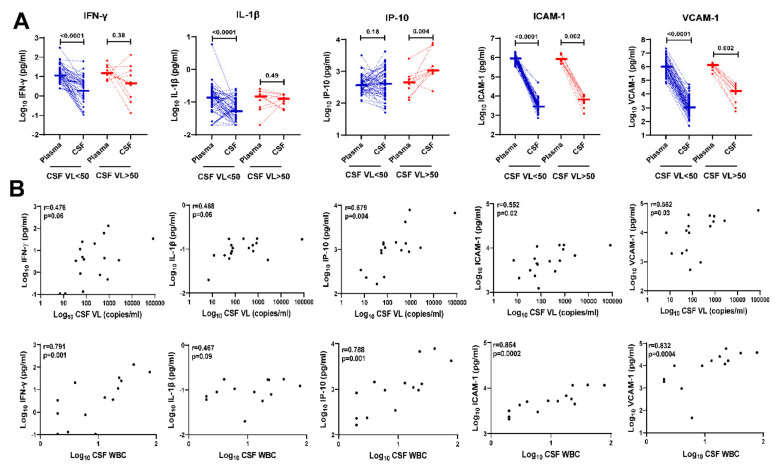
Upregulation of IFN-γ, IL-1β, IP-10, ICAM-1, and VCAM-1 in CSF of HIV+ individuals on ART with detectable CSF HIV RNA correlates with CSF HIV RNA copies/mL and CSF white blood cell count. (**A**) Comparison of IFN-γ, IL-1β, IP-10, ICAM-1, and VCAM-1 levels in paired plasma and CSF of HIV+ individuals on ART with CSF VL < 50 copies/mL (blue dotted lines, n = 60) or >50 copies/mL (red dotted lines, n = 10). *p*-values calculated using Wilcoxon signed-rank test; significant differences (*p* < 0.05) are indicated. (**B**) Correlation of log10 CSF IFN-γ, IL-1β, IP-10, ICAM-1, and VCAM-1 levels with detectable CSF HIV RNA copies/mL (top row, n = 17) and CSF WBC counts (bottom row, n = 14 after omitting 3 individuals with no CSF WBC). Correlation analyses included 4 additional time points from HIV+ individuals with CSF VL > 50 copies/mL and 3 HIV+ individuals with CSF VL < 50 copies who had detectable CSF HIV RNA (range, 7 to 35 copies/mL) measured by single-copy assays. Spearman’s rank correlation r and *p*-values are shown for each plot.

Next, we compared levels of IFN-γ, IL-1β, IP-10, ICAM-1, and VCAM-1 in paired plasma vs. CSF of HIV+ individuals on ART according to CSF VL < 50 copies/mL and >50 copies/mL (Figure 2A). IP-10 showed the most significant upregulation in CSF compared with plasma among HIV+ individuals on ART with CSF VL > 50 copies/mL (*p* = 0.004), while IFN-γ, IL-1β, ICAM, and VCAM-1 levels were higher in plasma compared with CSF among both HIV+ individuals with CSF VL < 50 or >50 copies/mL. In contrast to the upregulation of IP-10 in CSF vs. plasma observed among HIV+ individuals with CSF VL > 50 copies/mL, IP-10 levels were similar in CSF compared with plasma among HIV+ individuals with CSF VL < 50 copies/mL.

Previous studies have detected correlations between increased CSF HIV RNA copies/mL and/or increased CSF WBC and upregulation of CSF inflammatory biomarkers in HIV+ individuals with CSF viral escape [12,14,16,29,37,39,42]. Therefore, we addressed this question for CSF biomarkers that were upregulated in HIV+ individuals with CSF VL > 50 copies/mL vs. <50 copies/mL (Figure 1). In these analyses using Spearman’s rank correlation test, CSF HIV RNA copies/mL and CSF white blood cell count correlated with CSF IFN-γ (r = 0.476, *p* = 0.06; r = 0.791 *p* = 0.001, respectively), IP-10 (r = 0.679, *p* = 0.004; r =0.788, *p* =0.001), ICAM-1 (r = 0.552, *p* = 0.02; r = 0.854, *p* = 0.0002), and VCAM-1 (r = 0.562 *p* = 0.03; r = 0.832, *p* = 0.0004); IL-1β showed similar increasing trends (r = 0.468, *p* = 0.06; r = 0.467, *p* = 0.09) (Figure 2B).

### 3.4. CSF VL > 50 Copies/mL Is Associated with Increased YKL-40 But Not NFL and GFAP in CSF of HIV+ Individuals on ART

**Figure 3:**
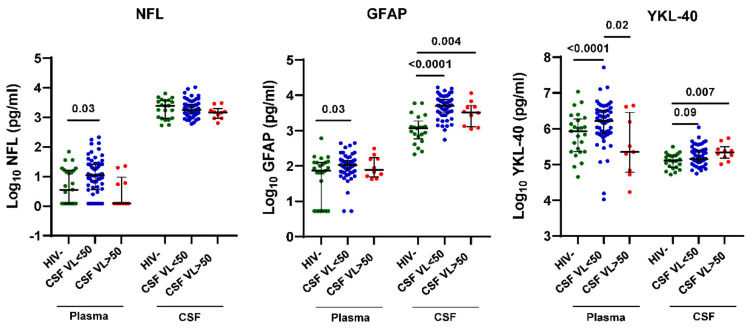
CSF VL > 50 copies/mL is associated with increased CSF YKL-40 but not NFL and GFAP in HIV+ individuals on ART. NFL, GFAP and YKL-40 levels in plasma and CSF from HIV+ individuals on ART stratified by CSF VL > 50 copies/mL vs. <50 copies/mL and HIV- controls. Horizontal bars represent medians, vertical lines the interquartile range (IQR). *p*-values calculated using Mann-Whitney U-test; significant differences (*p* < 0.05) between groups by CSF VL > 50 copies/mL vs. <50 copies/mL are indicated.

Given that some previous studies have detected increased CNS injury biomarkers in HIV+ individuals with CSF VL > 50 copies/mL, particularly those with associated neurological symptoms [15,18,28,31,40], we compared plasma and CSF NFL, GFAP, and YKL-40 levels between groups by HIV status or CSF VL > 50 copies/mL vs. <50 copies/mL (Figure 3). Plasma NFL, GFAP, and YKL-40 levels were increased in HIV+ individuals with CSF VL < 50 copies/mL but not CSF VL > 50 copies/mL compared with HIV- controls (*p* = 0.03, 0.03, and <0.0001, respectively). In contrast, CSF GFAP and YKL-40 levels were increased in both HIV+ individuals with CSF VL < 50 copies/mL and >50 copies/mL vs. HIV- controls (*p* < 0.0001, 0.004, 0.09, and 0.007, respectively), while CSF NFL levels showed no significant differences between groups. Among these CNS injury and glial activation biomarkers, CSF YKL-40 showed the most significant upregulation in HIV+ individuals with CSF VL > 50 copies/mL when contrasting differences between groups according to CSF VL > 50 copies/mL and <50 copies/mL vs. HIV- controls.

### 3.5. Inter-Relationship between CSF Inflammation Markers, Cell Adhesion Molecules, and Glial Activation Marker YKL-40 in HIV+ Individuals on ART

**Figure 4:**
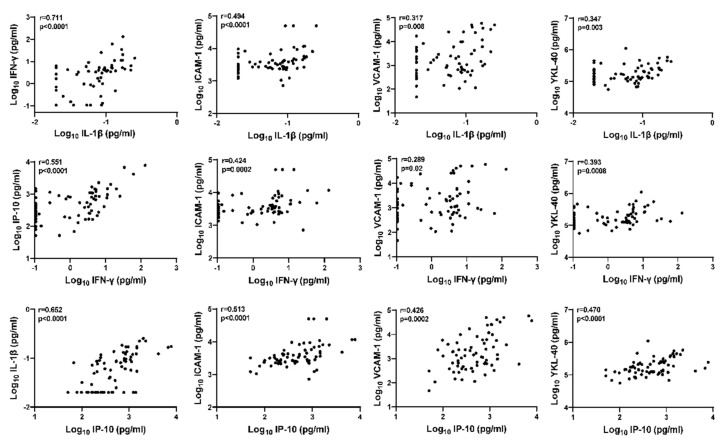
Inter-relationships between CSF IL-1β, IFN-γ, IP-10, ICAM-1, VCAM-1, and glial activation marker YKL-40 in HIV+ individuals on ART. Relationships between continuous variables (log10 transformed) were analyzed by Spearman’s rank correlation (n = 70); r and *p*-values are shown for each plot.

The preceding analyses suggest that the glial activation marker CSF YKL-40 may be upregulated in HIV+ individuals with asymptomatic CSF viral escape. Therefore, we evaluated inter-relationships between YKL-40 and other biomarkers of interest by Spearman’s rank correlation analyses (Figure 4). CSF YKL-40 correlated strongly with CSF IL-1β (*p* = 0.003), IFN-γ (*p* = 0.0008), and IP-10 (*p* < 0.0001). Additionally, CSF IFN-γ, IL-1β, and IP-10 were strongly correlated with each other (correlation r range: [0.551, 0.711], all *p* < 0.001). Both ICAM-1 and VCAM-1 were also correlated with IL-1β, IFN-γ, and IP-10 (correlation r range: [0.424, 0.513] and [0.289, 0.317], respectively; *p*-values range: ≤0.002 and [0.0002, 0.02], respectively). These findings suggest there may be functional inter-relationships between CSF IL-1β, IFN-γ, IP-10, ICAM-1, VCAM-1, and glial activation marker YKL-40 in the CNS of HIV+ individuals on ART that are upregulated in the setting of asymptomatic CSF viral escape.

### 3.6. Plasma and CSF YKL-40 Levels Are Elevated in HIV+ Individuals on ART with HAND Compared with No HAND or HIV- Controls and Correlate with Lower Global Neurocognitive T Scores and Higher NFL Levels

**Figure 5:**
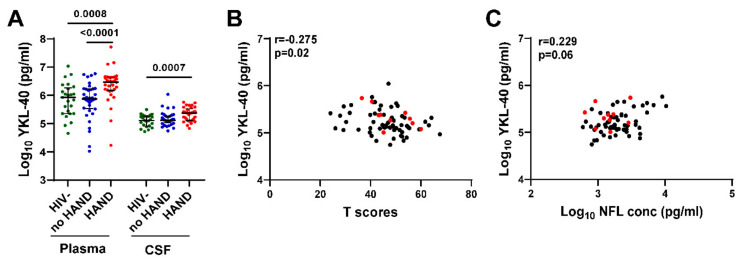
Plasma and CSF YKL-40 levels are elevated in HIV+ individuals on ART with HAND and CSF YKL levels correlate with lower global neurocognitive T scores and higher CSF NFL levels. (**A**). Plasma and CSF YKL-40 levels were compared between HIV+ groups with or without HAND and HIV- controls. Horizontal bars represent medians, vertical lines the interquartile range (IQR). *p*-values calculated using Mann–Whitney U test; significant differences (*p* < 0.05) are indicated. (**B**,**C**) CSF YKL-40 levels correlate negatively with global neurocognitive T scores (**B**) and positively with CSF NFL levels (**C**). Red dots indicate values for 10 HIV+ individuals with CSF VL > 50 copies/mL. Relationships between continuous variables were analyzed by Spearman’s rank correlation (n = 70); r and *p*-values are shown for each plot.

To evaluate the association between YKL-40 and neurocognitive status, we compared plasma and CSF YKL-40 levels between HIV+ groups with vs. without HAND diagnoses and HIV- controls (Figure 5A). Plasma YKL-40 level was significantly increased in HIV+ individuals with HAND compared with HIV- controls or HIV+ individuals without HAND (*p* = 0.0008 and *p* < 0.0001, respectively). In contrast, there was no significant difference in plasma YKL-40 level between HIV+ individuals without HAND and HIV- controls. CSF YKL-40 levels were significantly increased only in HIV+ individuals with HAND compared with HIV- controls (*p* = 0.0007), while there was no significant difference between HIV+ individuals without HAND vs. HIV- controls or HIV+ individuals with vs. without HAND. We did not detect significant differences in plasma/CSF NFL, GFAP, or other biomarkers between HAND vs. no HAND. CSF YKL-40 levels correlated negatively with global neurocognitive T scores (Spearman’s rank correlation r = −0.275, *p* = 0.02) and positively with CSF NFL levels (r = 0.229, *p* = 0.06) (Figure 5B,C), albeit weakly based on r and *p*-values. Together, these findings raise the possibility that increased CSF YKL-40, a marker of glial activation, may be involved in mechanisms leading to neuronal injury in some individuals with asymptomatic CSF viral escape.

## 4. Discussion

In this study of PWH on ART with vs. without asymptomatic CSF viral escape, in which groups with CSF VL > 50 copies/mL or <50 copies/mL were matched for age, duration of HIV infection, nadir CD4 count, and type of ART regimen, we showed that asymptomatic CSF viral escape was associated with increased IL-1β, IFN-γ, IP-10, ICAM-1, and VCAM-1 in CSF but not plasma. CSF IP-10 had the strongest association with CSF viral escape, consistent with previous studies [14,37]. Furthermore, CSF HIV RNA and WBC count correlated with CSF levels of IFN-γ, IP-10, ICAM-1, and VCAM-1. IFN-γ is mainly produced by activated T cells and induces IP-10, while IP-10 is the major chemokine attracting CXCR3 + CD4 + T cells into the CSF. Thus, our findings are consistent with the model recently proposed by Suzuki et al. [6] linking HIV RNA + CXCR3 + CD4 + T cells in CSF to brain injury in virally suppressed PWH on ART.

Our finding that IFN-γ and IP-10 levels correlate with HIV RNA levels and WBC counts in the CSF of PWH on ART is consistent with previous studies [13,14,16,49] and extends these observations by linking them to upregulation of other CSF biomarkers relevant for viral neuropathogenesis (i.e., IL-1β, ICAM-1, VCAM-1, YKL-40). Within the CNS, IP-10 is produced by activated myeloid cells, including monocytes, macrophages, and microglia, and astrocytes under inflammatory conditions. Increased IFN-γ in the CNS of PWH on ART has been linked to increased activated CD8+ T cells in CSF, which in turn induces immune activation of CNS myeloid cell populations and thereby contributes to an increased risk of neurocognitive impairment [3,49,50]. Although we did not detect an association between increased CSF IFN-γ or IP-10 levels and neurocognitive impairment in the present study, further study is warranted to address this question in larger prospective studies of PWH on ART with and without detectable CSF HIV RNA.

We found that CSF IL-1β, IFN-γ, IP-10, ICAM-1, and VCAM-1 levels were increased in PWH on ART with CSF viral escape and inter-correlated with each other, implying functional inter-relationships between these pro-inflammatory molecules. VCAM-1 and ICAM-1 are cell adhesion molecules that increase the attachment of circulating leukocytes to endothelial cells and thereby promote immune cell trafficking into the CNS. Within the CNS compartment, these adhesion molecules are expressed not only on endothelial cells that form the blood-brain barrier but also on epithelial cells in the choroid plexus that form the blood-CSF barrier. VCAM-1, ICAM-1, and other cell adhesion molecules are induced during CNS immune activation and neuroinflammation, which in turn increases immune cell trafficking across the blood-brain and blood-CSF barriers. Our finding that VCAM-1 is strongly upregulated in CSF viral escape is consistent with a previous study [37], and we link this finding to other neuroinflammatory mediators and potential mechanisms that contribute to CNS immune activation and pathogenesis in ART-treated PWH.

Although CSF NFL, GFAP, and YKL-40 levels were not significantly different between PWH with or without asymptomatic CSF viral escape, CSF YKL-40 correlated positively with CSF IL-1β, IFN-γ, IP-10, and NFL and negatively with neurocognitive T scores. Previous studies have suggested that CSF YKL-40 is associated with increased plasma and CSF HIV/SIV RNA, HIV/SIV encephalitis, and markers of axonal injury [19,21,22,48,51,52]. CSF YKL-40 is a biomarker of glial activation associated with neuroinflammation [19,21,53] that is produced by CNS macrophages, microglia, and astrocytes. YKL-40 is involved in neuroinflammation, tissue remodeling, and vascular disease [53,54]. While the role of CSF YKL-40 as a prognostic marker for HAND has been documented [19,21], its role in CSF viral escape in PWH has not been previously studied and warrants further evaluation. Together, these findings raise the possibility that the upregulation of YKL-40 may reflect indirect mechanisms linking the effects of viral transcription in the CNS to neuronal injury and cognitive impairment in ART-treated PWH.

As expected, PWH with vs. without asymptomatic CSF viral escape had more individuals (40%) with plasma VL > 50 copies/mL, a higher CSF VL, a lower CD4 count, and a higher CSF WBC, consistent with previous studies [14,16,26,27,29,36]. However, we detected no difference in the prevalence of HAND diagnoses, neurocognitive T scores, or BDI scores in PWH with vs. without asymptomatic CSF viral escape. We also found no difference in the CSF/plasma albumin ratio, an indicator of blood-brain barrier integrity. These findings are consistent with some previous studies that detected no significant differences in neurocognitive status or BDI scores in PWH on ART with undetectable plasma HIV RNA or low-level viremia and CSF viral escape [14,29,42].

We acknowledge several limitations of the study, including a relatively small sample size and small group of PWH with asymptomatic CSF viral escape, which reduces statistical power and the ability to detect some associations. Additionally, this was a cross-sectional study, so we could not evaluate the prognostic associations of biomarkers with future CSF viral escape and neurocognitive impairment. We did not have data on neurological signs or symptoms for the HIV- control group, which could have given a clearer picture of the potential value of some biomarkers. The viral load assays used to measure CSF HIV RNA levels had a detection limit of 50 copies/mL, so we could not evaluate associations between measurable CSF HIV RNA below 50 copies/mL and biomarkers. We did not have data to address some factors that impact durable viral suppression and the development of CSF viral escape, particularly ART adherence, treatment interruptions, viral blips, HIV genotypes, and drug resistance mutations. Additionally, we did not evaluate any macrophage- or microglia-specific markers. The majority of PWH were receiving outdated protease-inhibitor-based ART regimens (56%, majority on atazanavir or darunavir), and a minority were on an integrase inhibitor, which is now a mainstay of therapy (17%, with 10 of 12 taking raltegavir), so some findings may reflect the use of older ART drugs. Lastly, the studies were correlative, so we could not establish causality. Longitudinal studies of larger cohorts on newer ART regimens that incorporate sensitive single-copy assays to detect HIV below 50 copies/mL may overcome these limitations and determine the prognostic significance of CSF biomarkers.

## 5. Conclusions

In summary, we identified CSF biomarkers associated with asymptomatic CSF viral escape in PWH on ART in a matched-group cross-sectional study of HIV+ individuals with CSF viral escape and CSF HIV RNA > 50 copies/mL vs. <50 copies/mL. The studies identified a panel of CSF biomarkers reflecting inflammation and glial activation in the CNS that may serve as surrogate measures of viral persistence in the CNS and may be useful for future studies evaluating therapeutics targeting the CNS reservoir.

## Figures and Tables

**Table 1:** Demographic and clinical characteristics of the study cohort.

	HIV- Controls (n = 50)	HIV+ CSF VL < 50 Copies/mL (n = 60)	HIV+ CSF VL > 50 Copies/mL (n = 10)	*p*-Value
Age (years)	54 (50–60)	50 (46–55)	49 (44–61)	0.90
Male gender (n, %)	39 (78)	54 (90)	6 (60)	**0.03**
Race (n, %)				0.49
Black	12 (24)	20 (33)	2 (20)	
White	37 (74)	37 (62)	8 (80)	
Other	1 (2)	3 (5)	0 (0)	
Duration of HIV infection (years)		16 (11–22)	14 (8–23)	0.66
Plasma viral load > 50 copies/mL (n, %)		0 (0)	4 (40)	**<0.0001**
CD4 count (cells/μL)		512 (354–756)	318 (200–532)	**0.045**
Nadir CD4 count (cells/μL)		65 (13–192)	80 (14–147)	0.82
Hepatitis C seropositivity (n, %)		20 (33)	3 (30)	0.84
ART use (n, %)				0.38
Protease inhibitors (n, %)		34 (57)	5 (50)	
Integrase inhibitors (n, %)		11 (18)	1 (10)	
NNRTI (n, %)		25 (42)	3 (30)	
Duration of ART (years)		10 (7–13)	8 (7–11)	0.27
HAND diagnosis (n, %) ^Ϯ^		28 (47)	5 (50)	0.85
CPE score		8 (7–9)	8 (7–8)	0.35
CSF viral load (copies/mL)		40 (40–40)	156 (65–1364)	**<0.0001**
CSF white blood cells (cells/μL)		2 (0–4)	4 (0–22)	0.15
CSF protein (mg/dL)		37 (32–46)	29 (23–56)	0.47
CSF/plasma albumin ratio		4 (2–19)	6 (2–23)	0.91
BDI score		7 (1–18)	6 (3–10)	0.46
Global neurocognitive T score		47 (41–52)	47 (43–56)	0.57
Global clinical rating		4 (3–5)	4 (2–4)	0.40

Median (interquartile range) are shown unless otherwise indicated. *p*-values for comparisons between HIV+ groups with CSF viral load < 50 copies/mL vs. >50 copies/mL were calculated using Fisher’s exact or Chi-square test for categorical variables and Mann-Whitney U test for continuous variables. Bold font denotes *p* < 0.05. ^Ϯ^ HAND diagnoses among participants with CSF VL > 50 copies were all asymptomatic neurocognitive impairment (ANI) (n = 5) and among those with CSF VL < 50 copies were ANI (n = 18) or mild neurocognitive disorder (n = 10). Abbreviations: ART, antiretroviral therapy; BDI, Beck Depression Inventory-II; CPE, CNS penetration effectiveness; HAND, HIV-associated neurocognitive disorders; NNRTI, non-nucleoside reverse transcriptase inhibitors; VL, viral load.

## Data Availability

All data generated or analyzed during this study are included in the published article or available from the corresponding author on reasonable request.
